# DCs-based therapies: potential strategies in severe SARS-CoV-2 infection

**DOI:** 10.7150/ijms.47706

**Published:** 2021-01-01

**Authors:** Jian Han, Jiazhi Sun, Guixin Zhang, Hailong Chen

**Affiliations:** 1 General Surgery Department, The First Affiliated Hospital of Dalian Medical University, Dalian, China.; 2 Institute of Integrative Medicine of Dalian Medical University, Dalian 116044, China.; 3 Department of Pharmaceutical Sciences USF Health, Taneja College of Pharmacy University of South Florida, Tampa, FL, USA.

**Keywords:** dendritic cells (DCs) vaccine, Severe Acute Respiratory Syndrome Coronavirus 2 (SARS-CoV-2) infection, immunotherapy, immune system response, therapeutic vaccine

## Abstract

Pneumonia caused by the Severe Acute Respiratory Syndrome Coronavirus 2 (SARS-CoV-2) is spreading globally. There have been strenuous efforts to reveal the mechanisms that the host defends itself against invasion by this virus. The immune system could play a crucial role in virus infection. Dendritic cell as sentinel of the immune system plays an irreplaceable role. Dendritic cells-based therapeutic approach may be a potential strategy for SARS-CoV-2 infection. In this review, the characteristics of coronavirus are described briefly. We focus on the essential functions of dendritic cell in severe SARS-CoV-2 infection. Basis of treatment based dendritic cells to combat coronavirus infections is summarized. Finally, we propose that the combination of DCs based vaccine and other therapy is worth further study.

## Introduction

The attempts to treat virus infections occur even earlier than there was an understanding of the concept of a virus as an independent entity. The successful development and implementation to intervene prevention and treatment of virus infection over the past hundred years have had great advances on human and animal health. However, to this day, in the fight against virus infection, there is no real winner. In the past dozen years, the outbreak of the novel coronavirus is the third on record spillover of an animal coronavirus to humans after occurring of severe acute respiratory syndrome (SARS) started in 2002 and Middle East respiratory syndrome coronavirus (MERS-CoV) in 2014, which is resulting in a pandemic in global. This virus is named “SARS-CoV-2” from an international Committee on Taxonomy of Viruses and identified as the “sister virus of SARS”. There have been strenuous efforts to develop coronavirus therapy. Pneumonia caused by the SARS-CoV-2 is spreading continuously, the ability to adapt host and genomic mutation of Coronavirus (CoV) lead to hampering in treatment or control of the infection. At the time of this writing, more than 7 million COVID-19 cases are diagnosed worldwide, and it has been increasing still (https://www.who.int/emergencies/diseases/novel-coronavirus-2019). A minority of these cases progress to clinical forms which is with one or more severe complications, such as acute respiratory distress syndrome (ARDS) that is potentially fatal 1, 2. Thus, it is urgent to develop therapeutic strategies to face the SARS-CoV-2 viral infection at the severe stage.

The critical role of the immune system has been found in the discovery that there are reported high morbidity and mortality rates of human CoV infection in immunocompromised host as well as patients with comorbidities 3-6. In addition, it was reported that elderly patients with SARS-CoV-2 are at significant risk to develop severe disease and that the proportion of severe cases is elevated in hypertensive or diabetic patients with SARS-CoV-2 7. The research team recently observed infiltration of lymphocytes, monocytes and plasma cells in lung biopsy of a patient with SARS-CoV-2 pneumonia that is undergoing surgical operation of lung transplantation, which immunohistochemistry analysis also showed many CD3, CD4, CD8, CD20, CD79a, CD5 and CD38 positive cells. The expression of these cells is predominantly accumulated in the pulmonary interstitium 8. The result from another study in which an autopsy was performed in a patient that had died revealed that there was a large quantity of liquid overflow in patient's alveoli. On the other hand, it is reported that the IL-1β, IL-18, TNF-α, IL-6, IL-8 and IL-10 produced by immune cells causing a “cytokine storm” and overreaction of immune response related out‐of‐control events of inflammation, the latter would lead to serious organ damages including lung injury 9. These findings suggest that the SARS-CoV-2 mainly caused inflammatory response damaging alveolar and deep respiratory tract 10. Not only that, but the peripheral blood inflammatory factors are one of the “severe and critical clinical warning indicators”. It can be seen that the immune system could play a crucial role in pneumonia caused by SARS-CoV-2.

The basis for developing antiviral strategies or vaccine is a comprehensive understanding of the interaction between the immune system and viruses. This paper aims to find out the characteristics of SARS-CoV-2 as well as understanding of the interaction of this virus and the body immune system. In this review, we briefly outline the role of immune system in coronavirus infection and therapeutic potential of dendritic cells to treat coronavirus infection. We focus on strategies based on DCs (dendritic cells, DCs) and the efforts for the next generation of DC vaccine to combat Coronavirus infections. We talk about the research experience of SARS or MERS, which may help the design of DC vaccine. Finally, we summarized the clinical trials in patients with the viral disease using the therapeutic vaccine based on DCs as well as the unanswered questions.

## Characterization of the Coronavirus (CoV)

The family Coronaviridae contains four genera called *Alphacoronavirus*, *Betacoronavirus*, *Gammacoronavirus* and *Deltacoronavirus* 11. The members Coronavirus (CoV) is the largest known RNA viruses. The nucleocapsid (N), membrane (M), spike (S), and envelope (E) protein are the viral structural proteins expressed by CoV and they have been often associated with host reaction that stirs up an immune response by virus 12. Human HCoV-229E and HCoV-NL63 belong to the genus *Alphacoronavirus*, while HCoV-OC43, HCoV-HKU1, SARS-CoV, and MERS-CoV belong to genus *Betacoronavirus* 13.

Many have shown the importance of S protein of CoV because S protein makes CoV to achieve both survival and adaptive competence in varied hosts 14. Two functionally definite subunits, the S1 and S2 collectively constitute S protein of CoV. The former participates in receptor recognition, whereas the S2 subunit furthers membrane fusion. For SARS-CoV, the S protein is the potent target of neutralizing antibody. For MERS-CoV, the neutralizing humoral immunity is directed principally at the receptor-binding domain of the spike (S1 domain) protein. Further study on protein structure has clarified that both the N-terminal region and the C-terminal region of S1 can engage host receptors and thence function as receptor-binding domains (RBDs) 15.

In addition, CoV replicate only in convincing target tissues, this being predominantly the consequence of viral receptor distribution 16. The S-protein is the receptor-binding protein on the surface of CoVs and appears to be the essential determinant for infecting initial cross-species. Some receptors of CoVs have been found. Two human CoVs, HCoV -SARS, and HCOV -NL63 engage different regions of angiotensin converting enzyme 2 (ACE2) 17. HCoV-MERS engage to dipeptidyl peptidase 4 (DPP4) 18.

ACE2 protein is a transmembrane-peptidase which resides on the major cell surface of the epithelial, like cell type in the cardio-system, kidney, testes, lung and gastrointestinal tract. The internalization mechanism of S-protein binding to lipid rafts can also regulate the levels of the enzyme on the cell surfaces 19, 20. Previous studies have confirmed that SARS-CoV-2 also infects human alveolar epithelial cells through ACE2, and its pathogenic mechanism is very similar to that of SARS in 2002-2003. After attachment to a cellular receptor, viruses enter the cytoplasm by fusion with the plasma membrane 21. Therefore, it is of profound significance to study the mechanism of action of ACE2 receptor and SARS-CoV-2 for the development of drugs and vaccines for the treatment of diseases infected by SARS-CoV-2. Lately, Chinese scientists report the Cryo-EM structure of the full-length human ACE2 and a complex between the RBD of SARS-CoV-2 and ACE2. This has made important progress in understanding the SARS-CoV-2 infection in human cells, and may play a key role in promoting accurate diagnostic and treatment methods for SARS-CoV-2 pneumonia 22. Research just published these results open the door to design vaccine that can widely prevent SARS-CoV-2.

## DC background

As a special group, DCs have been found by German pathologist Paul Langerhans (1847-1888) more than a hundred years, but the absence of deep understanding causes of DCs to be ignored for a very long time. In the last decades, a series of studies by Silberberg and Steinman defined DCs as an antigen presenting cell by demonstrating that they may very well participate in immune responses 23. Importantly, DCs based vaccine to patients with prostate cancer received FDA approval in 2010 revitalized the field of study of DCs. Tremendous advances have been achieved in elucidating the function of DCs.

Single-cell RNA sequencing and epigenetic testing recently have distinguished the subsets of DCs by distinct function. Elucidating these characteristics is such as to illuminate that the functional specialization of DC subsets is related to their expression of surface receptors. In fact, determination of function and epigenetic signature of different DC subsets provides a rationale for immunotherapy.

Viruses and those that cells have been compromised by virus infection may carry some danger signals that could be recognized by DCs via receptors of the pattern recognition receptor family including Toll-like receptors (TLRs) and C-type lectin receptors (CLRs) 24. During the course of virus infection, in most cases, the Toll-like receptors (TLRs) activated MyD88 response and TRIF-dependent pathways, causing upregulation of IFN regulatory factors (IRF-3, IRF-5 and IRF-7) that are able to activate genes coding interferon and proinflammatory cytokines 25, 26. These give rise to the activation of other immune cells like NK cells, which move to the infection site. NK cells are the killer against virus in innate immune system 27. However, those cytokines that produced by mature DCs behavior including IL-12, IL-15, and IFN-α could modulate the function of NK cells 28, 29, which differ from those of “cytokine storm” mentioned above.

More significantly, DCs have a unique capacity* in vivo* to process antigens for presentation and do not infect DC themselves after engulfing of pathogen 30. On the contrary, DCs undergo maturation in processing pathogens. DC can present antigen in two ways: Peptide derived from proteins produced within the cell are displayed on the cell surface through binding to specialized antigen-presenting molecules termed MHC class I molecules. In a similar fashion, peptides produced by non-self-contained proteins are represented by MHC II molecules. Viral genome enters into the cell nucleus when DCs took over a virus. Newly synthesized peptides are marked by attachment to ubiquitin, degraded by proteasomes, and their fragments transported to the newly forming MHC class I molecule. The complexes are then transported to the cell surface for presentation and CD8 T cell response is triggered by MHC class I - bound antigens. In addition, dying virus-infected cells were engulfed by DCs 31. Exogenous antigens from the lysis are fragmented in endosomes and then attached to MHC class II molecule for transport to the cell surface 32, 33 and CD4 T cells can recognize MHC class II-bound antigens. Mature DCs are characterized by MHC presenting antigens, co-stimulatory molecules or co-inhibitory expression and more cytokines production. And they travel subsequently toward the secondary lymphoid organs to activate T cells 34. In secondary lymphoid organs, an immune response is characterized by the rapid proliferation of antigen-specific T cells after exposing to the antigens corresponding to their own receptors and become the main force to resist the virus. Ultimately, the T cell response is modulated by some of costimulatory and coinhibitory receptors that are expressed on the surface of DC. This process is very important for control of virus replication.

The receptor expressed by a developing thymocyte must be capable of binding with low-level affinity for some particular MHC self-molecule, either class I or class II, expressed by a resident thymic epithelial cell or APC. MHC class II restriction leads to CD4^+^ thymocytes and MHC class I restriction leads to CD8^+^ thymocytes, and then they leave to become mature CD4^+^ or CD8^+^ T cells. Hence, T cells in peripheral blood carry only one of CD4 or CD8. This differentiation makes the function of CD4^+^ or CD8^+^ T cells differently. T cells also must possess specific ability to recognize antigens, which has been realized by T cell receptors (TCRs). TCRs are heterodimeric protein complexes closed associated with either CD4 or CD8. That means the T cell must recognize the antigen fragment in association with an appropriate MHC molecule through its receptor complex: CD4 T cells called T “helper” cell recognize MHC class II-bound antigens, and CD8 T cells also called cell toxic lymphocytes (CTLs) recognize MHC class I-bound antigens. In secondary lymphoid organs, a significantly amount of CD8^+^ T cell (also called CTL) would proliferate after exposed to the antigens presenting by DC corresponding to their own receptors. They then can kill infected cells via perforin/granzyme-mediated lysis and death receptor-induced apoptosis. Not only that, activated CD4 T cells by three signals (antigen, costimulatory molecules, and cytokines) from dendritic cells involve the process that antibodies are produced by B lymphocytes in response to foreign antigens. It should be noted that the final player in the initial activation of CD8^+^ T cell is the “help” provided by CD4^+^ T cells specific for an antigen linked to the CD8^+^ T cell epitope 35.

The germinal centers in the secondary lymphoid tissues such as lymph nodes and spleen are the location where B cells encounter the antigen on follicular DCs and accept the help provided by Th cells (CD4^+^ T cells also called Th cells). Then those B cells will divide further and leave the center to form either plasma cells or memory cells.

**Figure 1:**
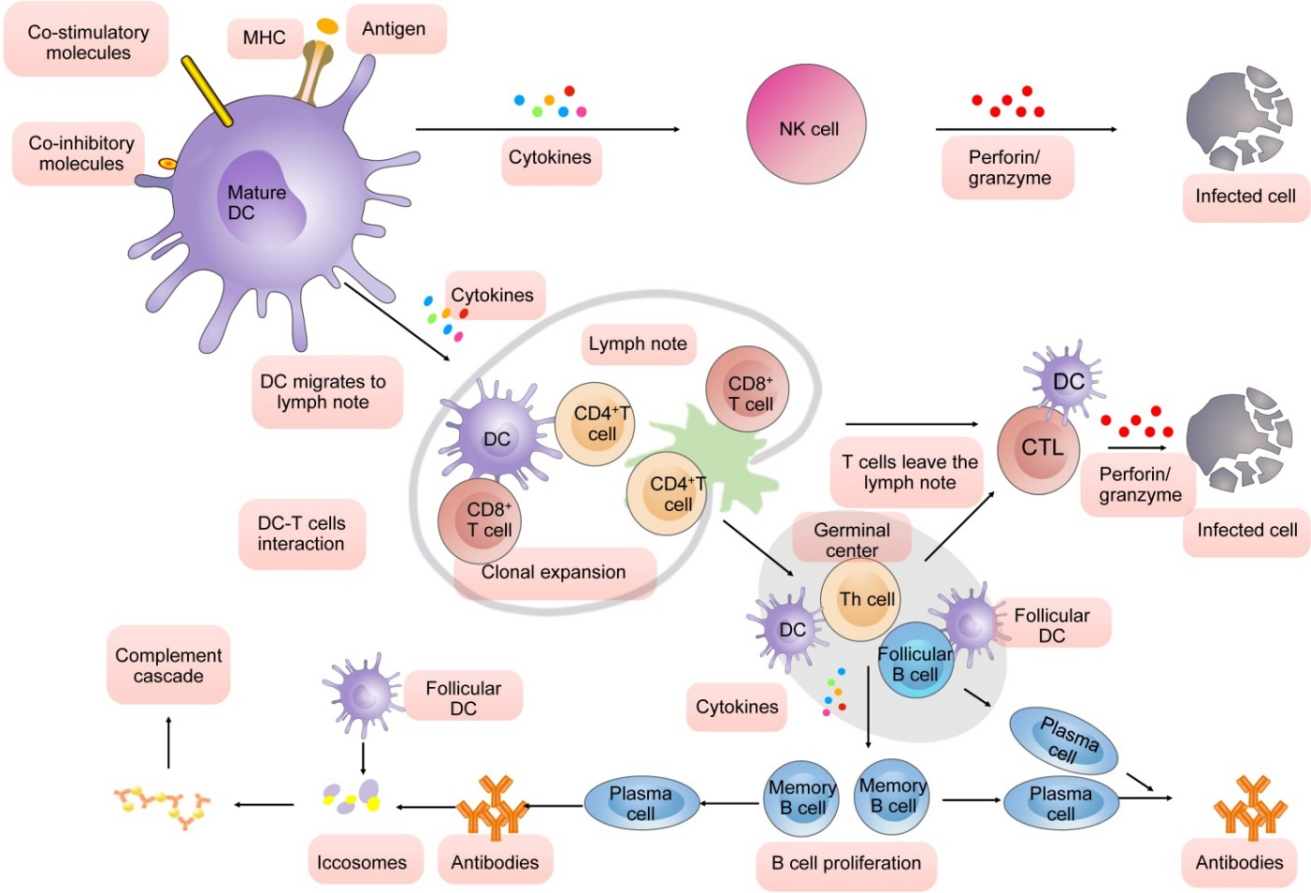
DC-mediated immune response in virus infection.

Besides these, in the unprimed animal, the dendritic cells simply provide a surface on which the antigen can be presented. In animals that have previously been exposed to antibody combine to form antibody-antigen complexes. The antigen-antibody complex (also called iccosomes) is required for full activation of the classical pathway of the complement cascade. The latter can lead to lysis of infected cells 36-38. In summary, dendritic cells (DC) play a pivotal role in virus infection (**Fig. 1**).

First, mature DCs are characterized by MHC presenting antigens, co-stimulatory molecules or co-inhibitory expression and more cytokines production. They produce cytokines to modulate the function of NK cells. NK cells can kill the infected cells. Second, mature DCs travel subsequently toward the secondary lymphoid organs to activate T cells through three signals (antigen, costimulatory molecules, and cytokines). A significantly amount of CD8^+^ T cell would proliferate after exposed to the antigens presenting by DC corresponding to their own receptors. They then can kill infected cells via perforin/ granzyme-mediated lysis and death receptor-induced apoptosis. Activated CD4^+^ involve the process that antibodies are produced by B lymphocytes in response to foreign antigens. It should be noted that the final player in the initial activation of CD8^+^ T cell (also called CTL) is the “help” provided by CD4 T cells specific for an antigen linked to the CD8^+^ T cell epitope. The germinal center in the secondary lymphoid tissues such as lymph nodes and spleen is the location where B cells encounter the antigen on follicular DCs and accept the help provided by Th cells (CD4^+^ T cells also called Th cells). Then those B cells will divide further and leave the center to form either plasma cells or memory cells. In animals that have previously been exposed to antibody combine to form antibody-antigen complexes (also called iccosomes). The iccosomes is required for full activation of the classical pathway of the complement cascade to promote phagocytic function.

## The defects of DCs in CoV infection

First, employing flow cytometry panels to measure account of innate immune cells found depletion of plasmacytoid dendritic cell (pDC) clearly correlated with disease severity 39. Investigators have also used single-cell RNA sequencing (scRNA-seq) to analyze the peripheral blood samples from patients with SARS-CoV-2 infection and found that the conventional dendritic cells (DCs) were significantly depleted in those who are acute respiratory distress syndrome (ARDS) 40. Another study is also performed investigating of immune cell including dendritic cell (DC) in convalescent patients infected SARS-CoV-2 and found not only depletion of dendritic cells but also reduction of functionality of maturation 41.

**Figure 2:**
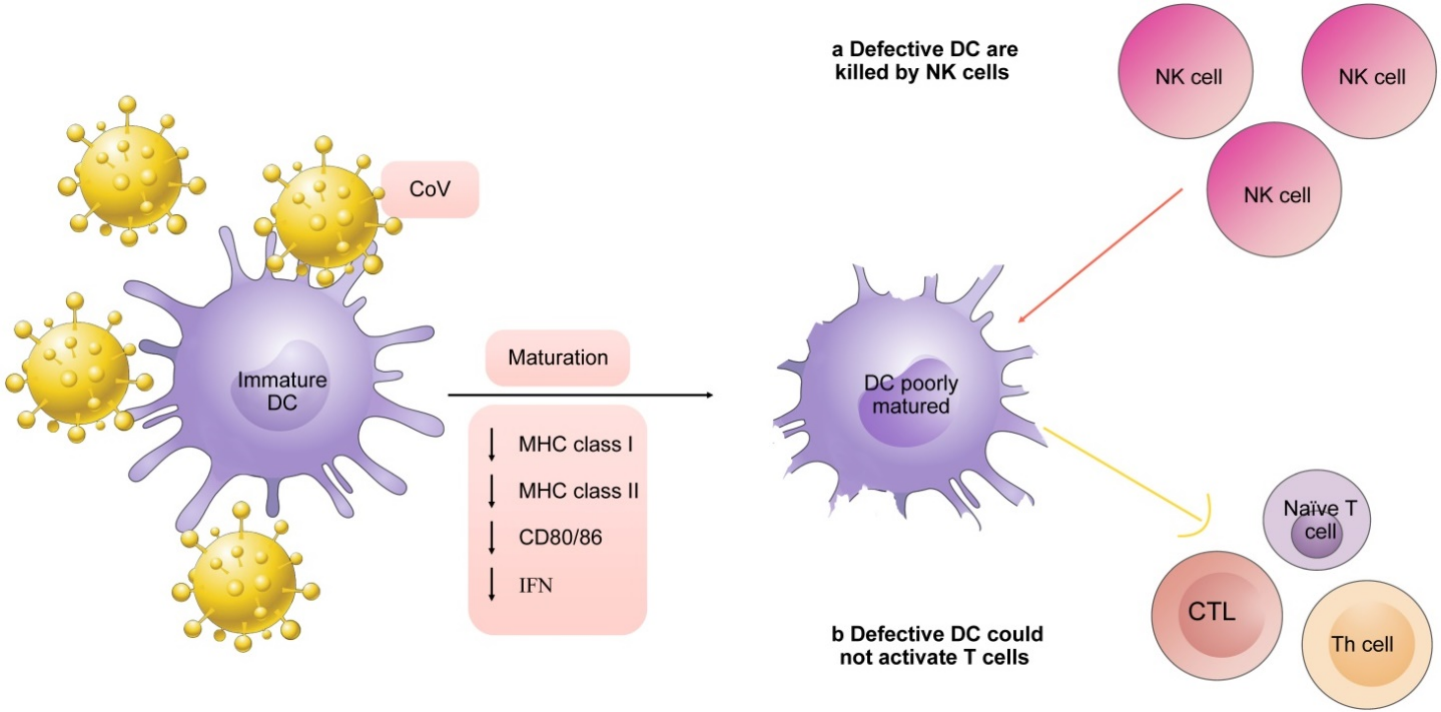
Defects of DCs in CoV infection. CoV infection enables DCs to downregulate expression of MHC-I, MHC-II, and CD80/86 as well as secretion of IFN. NK cells selectively induce apoptosis in those cells that do not exhibit MHC class I expression. Lack of mature signals of DCs results in inefficiency of T cell response.

To activate T cells, three characteristics from mature DCs are needed. One is the antigen presented by MHC molecules on the surface of DCs. The second one is mediated by costimulatory molecules or coinhibitory like CD80/86 on the surface of DCs. The third one is cytokines secreted by DCs 42. DCs are considered probable targets for CoV assault. A study found that APCs infected with MERS-CoV downregulates expression of MHC-I, MHC-II, and CD80/86. Lack of these DC signals results in inefficiency of T cell response to the virus. In addition, NK cells selectively induce apoptosis in those cells that do not exhibit MHC class I expression 43. In other words, some immature DCs are killed by NK cells in innate immune system before their homing. Thus, the continued production of impact on DCs due to viral persistence has a negative effect on NK cells, Th cells, and CD8 T cells activation (**Fig. 2**).

Second, specific virulence genes of CoV influence the cytokines production of DCs. Some expression of proteins on CoV appears to be antagonists of production or signaling of IFN 44. A study demonstrated that monocyte-derived dendritic cells (Mo-DCs) infected with MERS-CoV were in the absence of expression of IFN-β 45. Furthermore, the fact is the genes encoding HLA class II molecules of samples in patients with SARS-CoV-2, were down-regulated relative to the respective healthy controls 40.

## The advantages of mobilizing DCs for CoV therapy

Given DCs play a pivotal role in the struggle of the host cell in the restriction of infection by an intruding agent, we proposed that mobilizing DCs aims to induce both of antibody-mediated and cell-mediated immune responses might be crucial for SARS-CoV-2 vaccine. Generally, the advantages of utilizing DCs can be adopted in several aspects.

Firstly, results of the study suggested that MERS-CoV efficiently infected T cells from the peripheral blood and from human lymphoid organs, including the spleen and the tonsil through induced apoptosis in T cells 46. However, analysis of blood cells revealed that activated CD4 T cells and CD8 T cells were tested in of a convalescent patient with SARS-CoV-2 47. In patients that have recovered from SARS, T-cell responses have been shown in convalescence, and memory T-cell responses can be detected even for 6 years after infection 48. The experiments* in vitro* confirmed that virus-specific CD8^+^ T cells would locate in a position eliminating infected cells following primary SARS-CoV infection. They aptly produced multiple effector cytokines to reduce virus titers in the lung in mice infected SARS-CoV 49. Another study demonstrated that enhanced survival and reduced lung viral loads in SCID or BALB/c mice transferring SARS-CoV-immune splenocytes or *in vitro*-generated T cells 50. DCs can effectively induce CD4^+^ T cells to differentiate into Th1 CD4^+^ T cells secreting IFN-γ. And CD4^+^ T cells further help to secrete antibodies by B cells as well as antibody isotype switching. It was observed that the efficiency of specific immune response induced by DC-delivered peptides is 100-1,000 times more strongly than nonspecifically delivered peptides 51. Next, stimulations from mature DC reason to generate the function of CD8^+^ T cells. Moreover, mature DCs can induce T cell memory for both CD4^+^ and CD8^+^ responses 52, 53.

Also, DC as an antigen presentation carrier could effectively improve vaccine safety. Some researches supported that using an adenoviral-based SARS vaccine induced antibodies and T cell-mediated responses in monkeys immunized 54, which may cause harmful immune responses and inflammation 55-57. This is the reason contributing factor to the failure of viral vector-based vaccines. As a kind of DCs follicular dendritic cells are resident cells in the follicles of lymph nodes and splenic organ in human. They present antigen-antibody complexes to B lymphocytes and induce their proliferation and maturation as well as the immunoglobulin class switch. Neutralizing antibodies produced by B lymphocytes can neutralize viruses protecting the host cells against viral infection. As a target of neutralizing antibodies the receptor-binding domain (RBD) was identified in a study of MERS-CoV 58. We can transfect fragments of identified gene to find the region of antigenic peptide and screen out candidate peptides possessing acceptable amount of HLA-binding motifs. The peptides* in vitro* with higher capacity of binding to HLA are utilized for pulsing APCs to initiate specific response 59. Identification of antigens by the involvement of immune components is called “immunological”. Theoretically, using this we could mobilize DCs for CoVs therapy as a reasonably safe alternative.

## DC-based antiviral therapy: the clinical trial

Dendritic cell vaccination for virus disease was developed when it was observed that there was a significant increase of HIV-1-specific T cell response and proliferation in HIV-1-infected patients using the dendritic cell vaccine. In a clinical trial evaluating DC-based immunotherapy for human immunodeficiency virus type 1 (HIV-1)-infected patients combined antiretroviral therapy (ART), CD8^+^ T cell proliferative responses were observed in 4 out of the 10 subjects 60. Another phase II multicenter trial cleared that most of patients with HIV-1 infection received vaccinations of autologous dendritic cells following ART discontinuation were induced CD4^+^ and CD8^+^ T cell responses specific 61. By inducing specific cellular immune response particularly CTL response, could bring the destruction of infected cells in theory 62. Therefore, dendritic cell vaccination may be extended for other viral infectious diseases owing to their great merit and the potential for clinical response. A phase 1/2 clinical trial with monocyte-derived DC vaccine in SARS-CoV-2 infection was recently reported (ClinicalTrials.gov Identifier: NCT04386252). We will further follow-up to find out the evidence of safety and efficacy of appropriate of monocyte-derived DC vaccine in SARS-CoV-2 infection.

## Source and subsets of DCs in clinical trial

The CD14^+^ monocytes from peripheral blood mononuclear cells (PBMCs) of patients own are a major source of autologous Mo-DCs. Autologous Mo-DCs are have been widely used in the clinic as they are easier to be obtained 63. Notably, using Mo-DCs has to evaluate with caution due to modulation of monocytes function by SARS-CoV-2.

Generally, CD14^+^ monocytes can be further classified into three subsets: classical monocytes (CD14^++^CD16^-^), intermediate monocytes (CD14^+^CD16^+^), and nonclassical monocytes (CD14^+^CD16^++^) 64. Recent studies have shown that plasma concentrations of several inflammatory cytokines, such as granulocyte-macrophage colony-stimulating factor (GM-CSF), interleukin (IL)-6, tumour necrosis factor α (TNF-α), IL-2, 7, 10, and granulocyte colony-stimulating factor (G-CSF), were increased after SARS-CoV-2 infection, which not only decreases the number of monocytes but also perturb peripheral monocytes phenotypes and functions^65^-^67^. For example, it was found that the number of monocytes of patients with severe COVID-19 decreased, particularly of classical monocytes (CD14^++^CD16^-^), and that nonclassical monocytes (CD14^+^CD16^++^) significantly depleted in samples from COVID-19 patients with ARDS. Moreover, in patients with severe COVID-19, the frequencies of those CD14^+^CD16^+^ monocytes that have diminution of CD86, HLA class I and HLA-DR expression were found to be increased 39, 68, 69. Several down-regulation of type I interferon signaling and interferon stimulated genes (ISGs) including viral restriction factors and key receptor genes required for monocyte activation were found in patients with severe COVID-19^40^. Alternatively, data from different labs revealed that CD14^+^ monocytes could have been infected by SARS-CoV-2. For one thing infected CD14^+^ monocytes can remodel immune microenvironment, for another they can differentiate into macrophages 9, 70. As the efficacy of Mo-DCs vaccine closely relates to the quality of the patient-derived CD14^+^ blood monocytes, in the context of SARS-CoV-2 infection, there is the limitation of autologous Mo-DCs vaccine as strategy of preventive and therapeutic owing to the above reasons.

The rare CD34^+^ cells from hematopoietic stem cells (HSCs) as human DC precursors exist in the blood and bone marrow, which isolated and cultured with GM-CSF may differentiate into DCs *in vitro* 71. The umbilical cord blood (UCB) is rich in HSC, which can differentiate into DC precursor cells and became another source of generation of DCs* in vitro* 72. Cord blood has several potential advantages, including rapid availability and generally milder graft-versus-host disease (GVHD), leading to a reduced HLA-match requirement. Cord blood therefore became the preferred source of HSC-differentiated human DCs, with best-established culture protocols. It has been demonstrated that cryopreserved cord blood monocyte-derived DCs could induce allogeneic T cell proliferation 73, 74.

Bone marrow-derived MSCs (BM-MSCs) are ideal for cell-based therapy in various inflammatory diseases because of their immunosuppressive. Autologous BM-MSCs applications have some potential limitations, because auto-MSC extraction is time-consuming, making it difficult to use them promptly to treat acute diseases such as COVID. In contrast, allo-MSCs are readily available and can be administered immediately. In addition, commercial allo-MSC production should guarantee quality control and reduce the cost of cell therapies 75. Therefore, allo-MSCs are promising alternatives to auto-MSCs, with advantages with regard to time, cost, and quality assurance. Lineage Cell Therapeutics, Inc. toward the development of a potential vaccine against SARS-CoV-2 based VAC2 which is candidate platform that can product an allogeneic DC vaccine from human Embryonic Stem Cell (hESC) (https://lineagecell.com/). The platform technology that was comprised of allogeneic dendritic cells is predicted to change the landscape of DC vaccine. Therefore, research on stem cell and DC differentiation is still in its initial stages and face numbers of challenges. Extensive research to develop alternative strategies for this field is needed.

In addition, naturally occurring DCs without extended cytokines exposure stimulated the interest in targeting DCs *in vivo*. The administration of Flt3L induced a dramatic increase DC population *in vivo* provides convenience for naturally occurring DCs 76.

## The function of subsets of DCs

There are many subsets of DCs according to their functional and epigenetic features. Classical DCs (cDCs) and plasmacytoid dendritic cells (pDCs) are distinguished by their expression of CD1a and CD11c 77, 78. The cDCs terminally settle and develop to cDC1 and cDC2 subsets in the tissues after the pre-cDCs leave the bone marrow. Siglec-H-Ly6C- cells as direct progenitors of cDC1, whereas Siglec-H+Ly6C+ pre-cDCs were committed to the cDC2 lineage 79. The markers expression of pDCs includes CD45RA^+^, CD14^+^, Sirpa^+^, CD303^+^ (BDCA-2), CD304^+^ (BDCA-3), CD123^+^. Siglec-H-Ly6C^-^ cells as direct progenitors of cDC1 express hallmark transcription factors such as Id2 or BatF3, XCR1^+^, Clec9A^+^, BTLA^+^, Necl2^+^, CD141^+^ (BDCA-3). Recent studies shown that all of the cDC1 across tissues express the marker CD26^+^, Clec9A^+^ and Xcr1^+^ 80, which is not changing upon stimulation and migratory cDC1 cells are positive for CD103^+^ 81. While Siglec-H^+^Ly6C^+^ pre-cDC2 showed high Irf4 expression was committed to the cDC2 lineage 82. And the markers expression of cDCs2 also includes CD172a^+^ (Sirpa^+^), CD11b^+^, CD1c^+^ (BDCA-1), CD1a^+^, CD1b^+^, Clec10A^+^. cDC2s are related to the polarization of diverse subsets of CD4^+^ T cells 83.

The immune responses primed by subsets of DC determine the differentiation of naïve T cell to restrictive populations. Through the producing type I and type III IFNs, plasmacytoid DCs (pDCs) can promote innate immunity and CD8 T cell response 84-87. Through the secreting IL-12, cDC1s are able to activate CD8+ T cells 88 and to induce type 1 T helper cell (Th 1 cell) responses 89-91. Importantly, in the absence of IL-6 secretion, cDC1s convert T cells to Treg cells 92-94. In contrast, cDC2 cells excel in priming of Th2 or Th17 cells to fight against extracellular pathogens, fungi and allergy 95-97. Therefore, effector responses primed by pDCs and cDC1s may be an effective way to overcome the virus infection.

**Figure 3:**
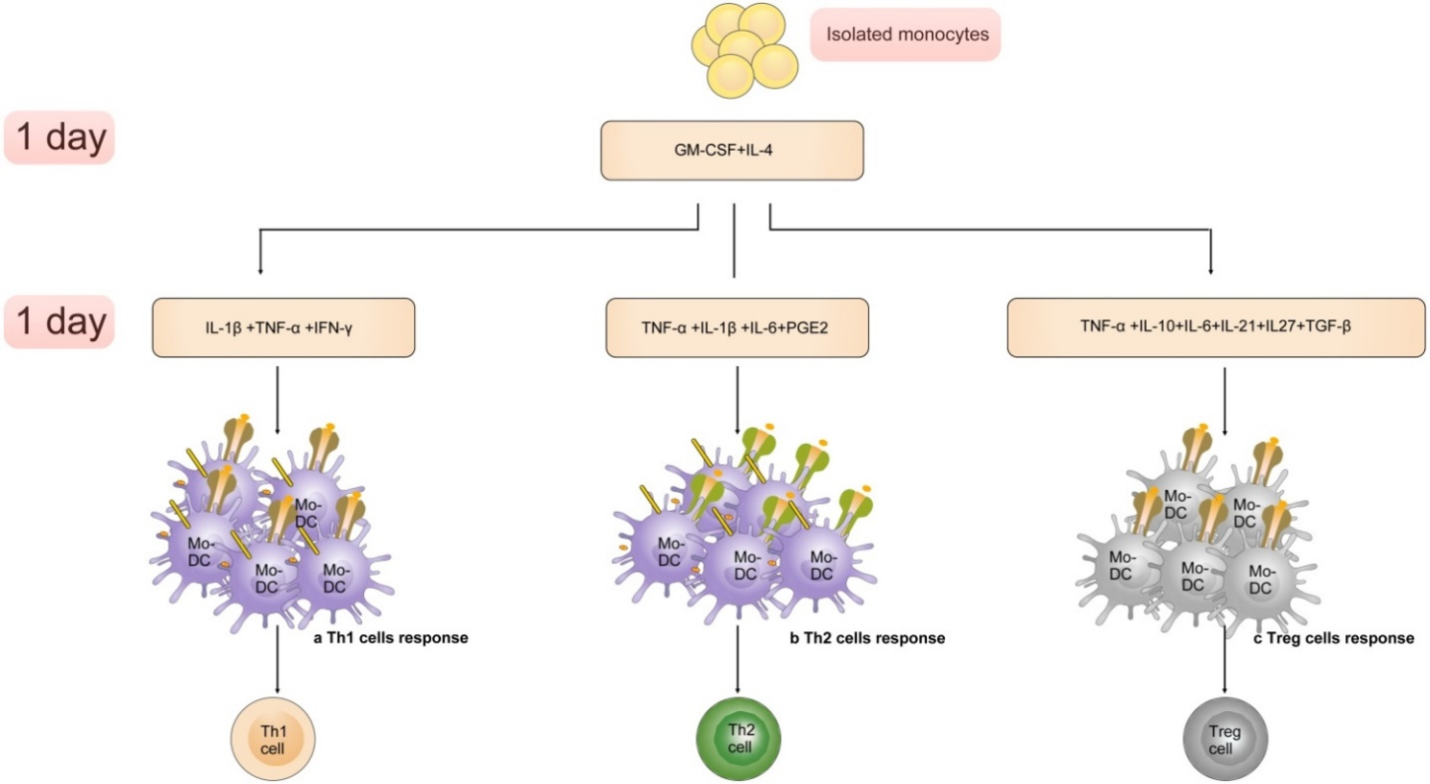
The “Fast DC” emerges and develops with the altered cytokine cocktails. A). Cultured with GM-CSF, IL-4 (1 day) and IL-1β, TNF-α, IFN-γ Mo-driven DC subsets are able to activate Th 1 cell response. B). The population produced through GM-CSF, IL-4 (1 day) and IL-1β, TNF-α, IL-6, PGE2(1 day) promote differentiation of CD4+ T cells towards a Th 2 cell phenotype. C). Cytokines including TNF-α, IL-10, IL-6, IL-21, IL27, TGF-β induce generation of DC subsets that promote the differentiation of Treg cell.

Altered cytokine cocktails induce generation of Mo-DC subsets, which are able to promote differentiation of CD4^+^ T cells towards a Th 1 cell, Th 2 cell or Treg cell phenotype 98 (**Figure 3**). The “Fast DC” that production of human DCs through GM-CSF+IL-4 (1 day) and IL-1β/TNF-α/IL-6/PGE2 (1 day) is exciting because it is an efficient and convenient method to manufacture 99, 100. Many studies focused on introducing of “Fast DC” into immunotherapy trials 101.

## Engineering antigen pulsed DCs

A key point in the design of the DC strategy for vaccine is antigen-bearing. In other words, the antigens can be loaded on DCs using some techniques making them become true immune stimulators. For introducing or “pulsing” antigens into DCs, various forms of antigen and delivery vehicle have been applied, including transfection of RNAs or immune- dominant peptides, and antibody-coated tumor cells, whole cell lysates, or necrotic cells.

### Antigen acquiring

The antigen acquiring is the primary condition for DCs to mount an antigen-specific immune response. The outbreak of the SARS-CoV-2 pneumonia is causing the global tensions. If the specific antigens are studied from beginning to end, this set of procedures will take a long time. The aid is too slow in being of any help. In addition, the binding domain of S protein receptor of 2019-SARS-CoV was 82% similar to that of SARS. Previously, scientists have made significant research on SARS, so we can refer to the research experience of SARS to quickly find potential antigens. Thus, the following existing vaccine research may help the design of antigen acquiring of DC vaccine.

First, structural proteins of SARS-CoV present important features in antigenicity of the virus, such like S protein 54, 102.

S protein induces not only the CD4 responses, it was also found many B cell epitopes in the S-RBD region 103-106. In fact, he fragments 450-650 of the S protein (S450-650) stand out from further research 107, 108. However, some SARS-CoV-specific neutralizing antibodies like m396, CR3014 can't bind SARS-CoV-2 spike protein 109. The fact speaks for a limiting of experience from previous studies. Further research demonstrated that the binding of SARS-CoV-2 neutralizing antibodies targeting the receptor-binding C-terminal domain 1 (CTD1s) of the S1 subunits related to the different conformational states of the SARS-CoV-2 surface spike 110.

Ongoing study on SARS-CoV-specific human monoclonal antibody CR3022 have led to the identification the ACE2 binding site within SARS-CoV-2 RBD that may be developed for reverse antigen acquisition.

A partial list of dominant epitope located the immunogenic regions of the SARS-CoV include a single immunodominant epitope S436 or S525 of the S protein, EP1 (amino acids 51-71), EP2 (134-208), EP3 (249-273), and EP4 (349-422) as well as N1 (QFKDNVILL) of the N protein, which could be used as a representative CTL antigen 49, 111, 112.

An article just published recently reported that the potential specific antigens of SARS-CoV-2 might be screened with the help of existing molecular databases and immunoinformatics, identified five CTL epitopes, three sequential B cell epitopes and five discontinuous B cell epitopes 113.

### Antigen loading

There are at present several approaches to “pulse” antigens into DCs. As mentioned, transfection of RNAs by utilizing lipid mediated transfection and electroporation are more effective but their drawback is the toxicity or loss of phenotype for DCs 114. Viral vectors and antigen nanoparticles (AgNPs) are modified and the disadvantages caused by above problems are solved 115, which has become an approved method to express antigen for DCs.

## Unanswered questions for human DC-based vaccine approaches

In conclusion, the expansion of autologous precursor cells or stem cells for the differentiation of DCs is time-consuming, which may be not suited to face acute inflammatory diseases. Allogeneic MSCs are a promising option because of their low immunogenicity and immunosuppressive. Thus, developing off-theshelf dendritic cell product would be an important step in treatment of covid-19 and the use of allogeneic MSCs (allo-MSCs) from donors is a reasonable strategy for antivirus. As with other novel therapies, there might be subtle clinical response and uncontrollable factors on DC-based vaccine field. Briefly these problems consist of DCs-related determination including maturity, quality, uniformity, safety, and standardization *ex vivo* manipulation (see **Figure A** for details). Some of these problems can be managed and involves determination of up-regulation of surface co-stimulatory molecules and cytokine production. Further studies are needed to address the problem of *ex vivo* manipulation, monitoring for quality control as well as insufficient pre-clinical and clinical data.

## Novel perspective: Targeting DCs

Although prior studies have characterized based on DCs vaccination strategies in great depth, including choice of antigen, expression vectors and delivery vehicle, they only represent the tip of the iceberg of potential value in DCs.

**Figure 4:**
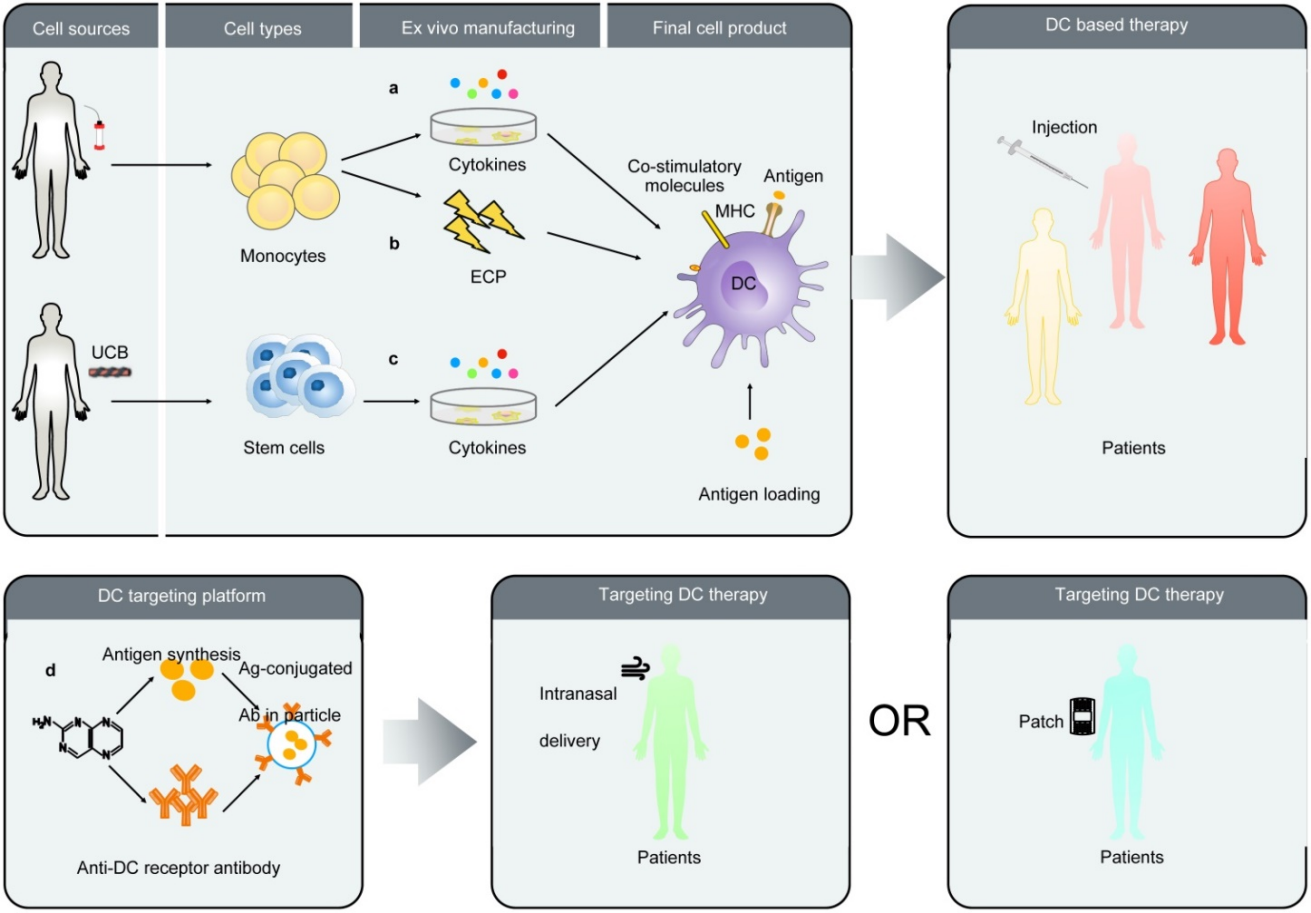
A summary of several DC-based vaccine strategies. **A.** The monocytes were obtained using CD14 beads isolated from blood. They then are cultured with cytokines to further derive functional DCs. After pulsing antigen these autologous DCs will be injected patient. **B.** There is another approach unlike described above. The Mo-DCs were obtained by extracorporeal photopheresis (ECP). **C.** The CD34^+^ isolated from UCB proliferating hematopoietic stem cells as a source of DC precursors have been developed. **D.** Employing a target antigen-fused anti-DC receptor antibody synthesize particles that targeted DCs in lymphoid tissues. The routes of administration include intranasal or by patch.

Targeting DCs *in vivo* and the function of distinct DC subsets is a hot topic issued in the present years. The DEC205 is an endocytic C-type lectin receptor expressed by murine and human DCs in different organs 116-118. Using this receptor, antigens may be targeted to DCs *in vivo*. New DC-targeting strategy may achieve antigen loading, DC maturation, enrichment, and presentation *in vivo* via encoding a fusion protein comprised of an antigen and a single-chain Fv antibody (scFv) specific for the DC endocytic receptor DEC205. It is impressive for patients with SARS-CoV-2 infection that using this platform develops a DC-targeting vaccine, which might enhance antigen specific CD8^+^ T cell immune response 119. The function of the DC-targeting platform has also been supported by the results of studies in other viral infectious disease model such as HIV and plague. Human DEC-205 targeted antibody (MG38.2 Ab) 117 have acted as a vaccine against Epstein-Bar-virus (EBV) primary infection. The extracorporeal photopheresis (ECP) represents another novel strategy of generating of DCs 120. Exposing extracorporeally circulated blood to lethal doses of the photoactivatable DNA-crosslinking drug induced monocyte to mature into DC that possess antigen specificity and ability to control disease. Researchers also plan to improve the preventive vaccine via preparative ECP-driven DCs that were loaded microbial antigen, such as killed virus. Owing to its pleasant safety profile, this approach can be successfully used to immunotherapy in all area 121. Some of extracellular vesicle (EV) studies suggests that the EVs secreted by DCs still attract researchers' attention due to its ability to activate T cells responses and promote humoral responses. Particularly, different stimuli induced changes of DCs with the variety of extracellular vesicles secreting by DCs 122. The success of such efforts opened some new perspectives in design of antiviral therapy based on DCs. **Figure 4** is a summary of several DC-based strategies.

## Discussion

For acute virus infection, it is unlikely that based-DCs strategies are used as a single therapeutic. In fact, combination therapy may be more effective. The past two decades have witnessed the emergence of several potential, but fundamentally different strategies for virus infection, including vaccine, monoclonal antibody, peptide, interferon therapy, oligonucleotide (AO)-based methods and relevant small molecule drugs. Numerous compounds are now undergoing clinical trials. In 2004 and 2007, American researchers found three kinds of monoclonal antibodies that can block the infection of SARS-CoV. In 2013 Japanese researchers found that the humanized preparation of ys110 monoclonal antibody has been used in clinical trials for other indications, and will become a promising candidate drug for the treatment of MERS-CoV in the future. In 2014, researchers from China and the United States identified three monoclonal antibodies targeting MERS-CoV spike glycoprotein receptor from large number of candidate antibody libraries. The Food and Drug Administration (FDA) issue an Emergency Use Authorization (EUA) for emergency use of remdesivir for the treatment of hospitalized 2019 coronavirus disease (COVID-19) patients. Antivirus therapy may accelerate infected cells death, which becomes new antigen source for DCs *in vivo*. Such hypothesis predicts that it is possible to combine multiple therapies with different mechanisms for improving anti- SARS-CoV-2 in greater numbers of patients.

It is worth mentioning here that the andrographolide as a major component of anti-SARS-CoV-2 Chinese traditional medicines 123. Chinese traditional medicines have shown the functions of clearing heat and anti-inflammatory in patients with SARS-CoV-2^7^, ^123^. Some of them were selected into the Guidelines for the Diagnosis and Treatment of Novel Coronavirus Infection (Trial Version 7) published by the National Health Commission of the People's Republic of China (NHCPRC). The andrographolide is one of essential compounds used for promoting tolerogenic DCs by blocking nuclear factor-kappa B (NF-kB) activity 124. Tolerogenic DCs may promote regulatory T cell (T_reg_ cell) activation. In contrast to other immunotherapies, T_reg_ cell strategy could show advantages for the harmful immune response induced by viral infection 52,125. Under these circumstances, tolerogenic DCs strategy may also be beneficial. This new field has become an emerging topic of more and more research attention. Hence, further studies regarding the effects of Chinese traditional medicine on function of DCs are worth.

The harmful immune response such as “cytokine storms” appearing in patients with infection of SARS-CoV-2 have reported 126. The importance of using immunotherapeutics have been reviewed 127. Another aspect worth noting refers to that the combination of cell therapy and cytokine blockade therapy is important. Nevertheless, it has not yet gained enough attention. At this stage, combination therapy may be helpful to patients.

## Figures and Tables

**Figure A:**
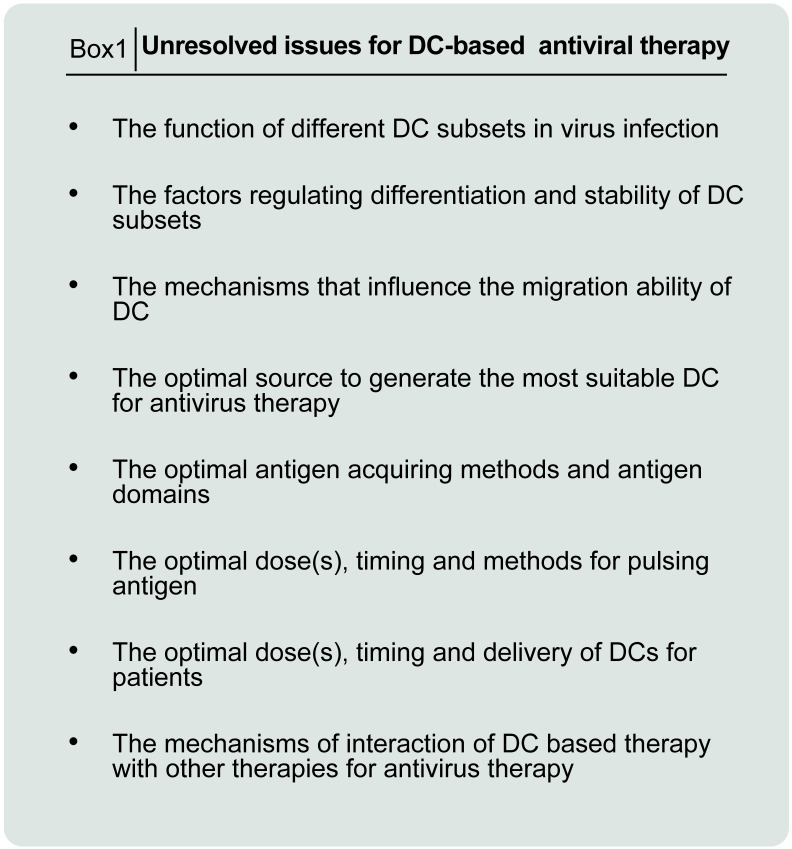
unresolved issues for DC-based antiviral therapy.
